# The place of vericiguat in the landscape of treatment for heart failure with reduced ejection fraction

**DOI:** 10.1007/s10741-021-10146-1

**Published:** 2021-07-21

**Authors:** Alberto Aimo, Vincenzo Castiglione, Giuseppe Vergaro, Giorgia Panichella, Michele Senni, Carlo Mario Lombardi, Michele Emdin

**Affiliations:** 1 Institute of Life Sciences, Scuola Superiore Sant’Anna, Piazza Martiri della Libertà 33, Pisa, Italy; 2 Cardiology Division, Fondazione Toscana Gabriele Monasterio, Piazza Martiri della Libertà 33, Pisa, Italy; 3 Department of Medical and Surgical Specialties, Radiological Sciences, and Public Health University and Civil Hospital, Brescia, Italy; 4 Cardiovascular Department & Cardiology Unit, ASST Papa Giovanni XXIII, Bergamo, Italy

**Keywords:** Heart failure, Soluble guanylate cyclase, Cyclic guanosine monophosphate, Treatment, Vericiguat

## Abstract

The significant morbidity and mortality associated with heart failure with reduced (HFrEF) or preserved ejection fraction (HFpEF) justify the search for novel therapeutic agents. The nitric oxide (NO)–soluble guanylate cyclase (sGC)-cyclic guanosine monophosphate (cGMP) pathway plays an important role in the regulation of cardiovascular function. This pathway is disrupted in HF resulting in decreased protection against myocardial injury. The sGC activator cinaciguat increases cGMP levels by direct, NO-independent activation of sGC, and may be particularly effective in conditions of increased oxidative stress and endothelial dysfunction, and then reduced NO levels, but this comes at the expense of a greater risk of hypotension. Conversely, sGC stimulators (riociguat and vericiguat) enhance sGC sensitivity to endogenous NO, and then exert a more physiological action. The phase 3 VICTORIA trial found that vericiguat is safe and effective in patients with HFrEF and recent HF decompensation. Therefore, adding vericiguat may be considered in individual patients with HFrEF, particularly those at higher risk of HF hospitalization; the efficacy of the sacubitril/valsartan-vericiguat combination in HFrEF is currently unknown.

Heart failure (HF) affects over 20 million people worldwide, and its prevalence is increasing because of the population ageing and the better outcome after acute cardiovascular events [1]. Many therapeutic options are now available for HF with reduced EF (HFrEF). First, the success of early clinical trials established angiotensin-converting enzyme inhibitors (ACEi), angiotensin receptor blockers (ARBs), beta-blockers, and MRA as the foundation for drug treatment of HFrEF [1, 2]. Afterwards, ivabradine [3] and, most importantly, sacubitril/valsartan [4] were added to the list of disease-modifying therapies. Finally, sodium-glucose cotransporter 2 inhibitors (SGLT2i) empagliflozin [5] and dapagliflozin [6, 7], the cardiac-specific myosin activator omecamtiv mecarbil [8], and the soluble guanylate cyclase (sGC) stimulator vericiguat [9] were found to improve outcomes in HFrEF even on the background of a standard therapy with ACEi/ARB, beta-blockers, and MRA. Among the newer therapies, sacubitril/valsartan modulates the neurohormonal imbalance characteristic of HF by acting directly on the natriuretic peptide system. All the other drugs have different mechanisms of action, with vericiguat directly acting on cGMP second messenger, SGLT2i, targeting the kidney and possibly also the heart [10], and omecamtiv mecarbil enhancing cardiac inotropism without increasing oxygen consumption or calcium levels in cardiomyocytes [11].

The emerging challenge of HFrEF treatment is to tailor the therapeutic strategy on each individual patient, namely to identify the combination of treatments with the greatest benefit (in terms of survival, but also quality of life) while minimizing the adverse events (AEs). In this review, we analyze current evidence about vericiguat trying to identify its place in the changing landscape of HFrEF treatment.

## Mechanism of action of therapies targeting the NO-sGC-cGMP pathway

Nitric oxide (NO) is produced by endothelial NO synthase (eNOS), which is induced by laminar flow and shear stress [12]. NO diffuses to neighbouring cells and binds to the haeme group of sGC, which synthesizes cGMP, then activating protein kinase G (PKG). Proteins phosphorylated by PKG in the heart and vessels promote diastolic relaxation; improve coronary blood flow; inhibit the development of inflammation, hypertrophy, and fibrosis in response to cardiac damage; and improve ventricular-arterial coupling [13]. The heart expresses 7 phosphodiesterase (PDE) isoforms, which inactivate cGMP to GMP [14]. PDE3 inhibitors, such as milrinone and enoximone, are used in acute HF (AHF), and PDE5 inhibitors, such as sildenafil and udenafil, improve contractile function in systolic HF, blunt left ventricular (LV) hypertrophic remodelling, reduce myocardial infarct size, and suppress ventricular arrhythmias [15], although both classes do not ameliorate the outcome of HF patients [16]. Finally, natriuretic peptides (NPs), most notably atrial NP (ANP) or B-type NP (BNP), exert their biological effects through transmembrane receptors (NR-A and NR-B) with GC activity (particulate GC, pGC) [17], while NR-C receptors act as clearance receptors, decreasing plasma NP concentration, together with enzymatic cleavage by vasopeptidases like neprilysin (NEP) [18].

In HFrEF, impaired LV systolic function leads to tissue hypoperfusion, which causes inflammation and oxidative stress, leading to decreased NO bioavailability and cGMP deficiency [19]. The cGMP deficiency indeed has deleterious effects on the heart, kidneys, and vessels (including the pulmonary circulation), which may contribute to HF progression [13]. A blunted response to NPs is commonly observed in HFrEF, which may be due to several mechanisms including altered production or clearance of active NPs, their binding to membrane receptors, or intracellular effects [17]*.* By acting on a downstream target of the NO-sGC-cGMP pathway, sGC modulators may circumvent NP resistance more effectively than other therapeutic strategies aiming to enhance NP concentration, such as the administration of pharmacological doses of recombinant BNP (nesiritide, ularitide), associated with worsening renal function and no effect on outcome.

The sGC activator cinaciguat increases cGMP levels by direct, NO-independent activation of sGC, with a high risk of hypotension [20]. Conversely, sGC stimulators enhance sGC sensitivity to endogenous NO, which possibly explains their neutral effects on blood pressure. While the sGC stimulator riociguat has a shorter half-life requiring 3 administrations per day [21], vericiguat has a more favourable pharmacology.

## Pharmacology of vericiguat

Vericiguat is a weakly basic drug with a low water solubility and high intestinal permeability (class II according to the Biopharmaceutics Classification System) [22]. In healthy humans, vericiguat (≤ 10 mg, immediate-release [IR] tablets) is rapidly absorbed (median time to reach maximum plasma concentration ≤ 2.5 h in the fasted state) with 18–22 h half-life. Pharmacokinetic studies reported no deviation from dose proportionality or unexpected accumulation [23]. Administration of vericiguat 5 mg IR tablets with food increases bioavailability up to 93% (about 19% increase compared to fasted state), reduces pharmacokinetic variability, and prolongs absorption relative to the fasted state [23]. Vericiguat has about 98% plasma protein binding, with serum albumin being the main carrier, and is a low-clearance drug (1.6 L/h in healthy volunteers and 1.3 L/h in patients with HFrEF) [27]. Beyond the titration regimen, no further dose adjustment is recommended in patients with estimated glomerular filtration rate (eGFR) down to 15 mL/min/1.73 m^2^ or those with mild-to-moderate liver disease [9]. In healthy human subjects, 53% and 45% of an administered dose is excreted via the urine and faeces, respectively. The main metabolic pathway of vericiguat is glucuronidation via uridine diphosphate-glucuronosyltransferase (UGT) 1A9 and 1A1 [24, 25]. Further in vitro studies with human liver microsomes indicate that vericiguat has no inhibitory effects on the major cytochrome P450 (CYP) isoforms [24], UGT isoforms, or major transport proteins [25]. This denotes a small potential for pharmacokinetic interactions, as confirmed by 10 phase 1 studies searching for possible interactions with drugs that affect intestinal pH and NO signalling, inhibit or induce metabolic pathways, or common cardiovascular drugs [25].

## Vericiguat in HFrEF: clinical trials

In the setting of HFrEF, vericiguat has been investigated in phase 2 (SOCRATES-REDUCED) and phase 3 trials (VICTORIA) (Table 1). Both these trials focused on patients with a high risk of decompensation, where the diuretic and natriuretic effects achieved by sGC stimulation were expected to produce the greatest benefit.

**Table 1:** Efficacy and safety of vericiguat: evidence from clinical trials

Study^ref^	Setting	Patient n	Treatment arms	Efficacy	Safety
SOCRATES-REDUCED [26]	HF, LVEF < 45%, BNP ≥ 300 ng/L (≥ 500 ng/L if AF) or NT-proBNP ≥ 1000 ng/L (≥ 1600 ng/L if AF), < 4 weeks from HF decompensation	351	Vericiguat (1.25 mg, 2.5 mg, 5 mg, 10 mg daily) for 12 weeks vs. placebo	Pooled vericiguat vs. placebo: no significant difference in Δlog(NT-proBNP) from baseline to week 12 (*p* = 0.15)	Any AE: 71.4% vericiguat 10 mg, 77.2% placebo
VICTORIA (9)	HF, NYHA II-IV, LVEF < 45%, BNP ≥ 300 ng/L (≥ 500 ng/L if AF) or NT-proBNP ≥ 1000 ng/L (≥ 1600 ng/L if AF), HF hospitalization < 6 months or worsening HF requiring iv diuretics < 3 months	5050	Vericiguat (target dose 10 mg daily) vs. placebo	Primary endpoint (CV death or HF hospitalization): HR 0.90 (0.82–0.98) HF hospitalization: HR 0.90 (0.81–1.00) Death or HF hospitalization: HR 0.90 (0.83–0.98)	Symptomatic hypotension: 9.1% vericiguat vs. 7.9% placebo (*p* = 0.12) Syncope: 4.0% vs. 3.5% (*p* = 0.30) Anaemia: 7.6% vs. 5.7% (serious AEs in 1.6% vs. 0.9%)

*6MWD* 6-min walking distance, *AE* adverse event, *AF* atrial fibrillation, *BNP* B-type natriuretic peptide, *BP* blood pressure, *CI* confidence interval, *CV* cardiovascular, *HF* heart failure, *HR* hazard ratio, *KCCQ-CCS* Kansas City Cardiomyopathy Questionnaire Clinical Summary Score, *KCCQ-CCS* Kansas City Cardiomyopathy Questionnaire Physical Limitation Score, *LAV* left atrial volume, *LVEF* left ventricular ejection fraction, *NT-proBNP* N-terminal pro-B-type natriuretic peptide, *NYHA* New York Heart Association, *SOCRATES-REDUCED* SOluble guanylate Cyclase stimulatoR in heArT failurE patientS with REDUCED EF, *VICTORIA* Vericiguat Global Study in Subjects with Heart Failure with Reduced Ejection Fraction

SOCRATES-REDUCED enrolled 456 patients with LV ejection fraction (LVEF) < 45% and a recent episode of HF decompensation, defined by worsening HF symptoms requiring hospitalization or outpatient administration of intravenous diuretics, signs of congestion, and elevated NP levels (BNP ≥ 300 ng/L or NT-proBNP ≥ 1000 ng/L; for patients in atrial fibrillation [AF], BNP ≥ 500 ng/L or NT-proBNP ≥ 1600 ng/L), excluding those with eGFR < 30 mL/min/1.73 m^2^ and systolic blood pressure < 110 or ≥ 160 mmHg. Patients were randomized to 5 arms (target maximal doses of 1.25 mg, 2.5 mg, 5 mg, and 10 mg once daily or placebo). Only 77% of patients completed the 12-week follow-up, and 72% of patients randomized to the highest dose reached the target of vericiguat 10 mg daily. The change in log-transformed N-terminal fraction of pro-BNP (NT-proBNP) over 12 weeks did not differ significantly in the pooled vericiguat group and the placebo arm, while the exploratory comparison between vericiguat 10 mg and placebo achieved statistical significance (*p* = 0.048). Patients on the highest vericiguat dose displayed also a greater increase in LVEF (*p* = 0.02). Vericiguat therapy did not seem to affect haemodynamic function and appeared safe, with lower rates of serious AEs than placebo. A similar proportion of AEs was observed in the pooled vericiguat group and the placebo arm [26]. A post hoc analysis showed that vericiguat treatment was also associated with decrease in plasma high-sensitivity C-reactive protein and serum uric acid. This effect was dose-dependent and most prominent in the highest dose group [27].

The VICTORIA trial enrolled patients with LVEF < 45%, a HF decompensation requiring hospitalization over the previous 6 months, and/or intravenous diuretics < 3 months [9, 28] and elevated circulating NPs (the same cutoffs as in SOCRATES-REDUCED trial) [29]. A total of 5050 patients were enrolled (76% men; 60% of patients were on triple medical therapy with a beta-blocker; a MRA; and either an ACEi, an ARB, or a sacubitril-valsartan [10%]) [9, 28], and randomized to vericiguat 2.5 mg once daily, up-titrated to 5 mg and then 10 mg at 2-week intervals, or placebo. Over a median 10.8-month follow-up, patients on vericiguat had a lower incidence of cardiovascular death or first HF hospitalization (hazard ratio [HR] 0.90, 95% confidence interval [CI] 0.82–0.98; *p* = 0.02), with a number needed to treat of around 24. These results were driven by a lower incidence of first HF hospitalization (HR 0.90, 95% CI 0.81–1.00), associated with a reduced number of HF hospitalizations (HR 0.91, 95% CI 0.84–0.99; *p* = 0.02). Vericiguat seems less effective in patients in the highest quartile of NT-proBNP levels (> 5314 ng/L), those aged ≥ 75 years, with worse renal function (eGFR 15–30 mL/min/1.73 m^2^), or LVEF 40–45%, although the trial was underpowered for these subgroup analyses [9]. The rates of symptomatic hypotension or syncope did not differ significantly between patients on vericiguat or placebo. Anaemia developed more often in patients on vericiguat than placebo, although it was rarely classified as a serious AE. Overall, serious AEs occurred in a similar proportion of patients in the 2 groups. Drug tolerability was further confirmed by an 89% rate of target dose achievement [9].

In some post hoc analyses, the superior efficacy of vericiguat over placebo was confirmed for NT-proBNP levels at randomization up to 8000 ng/L [30]. Furthermore, vericiguat reduced the primary endpoint in all subgroups identified by the time from prior HF hospitalization (less than 3 months, 3 to 6 months, or need for intravenous diuretics < 3 months without hospitalization). Even the safety profile of vericiguat did not change across these categories [31]. Therefore, vericiguat prescription does not seem to require further patient phenotyping beyond the inclusion and exclusion criteria of the VICTORIA trial.

## Vericiguat in HFrEF: indirect comparisons with other drugs

While a comparison of HR values for the primary and secondary endpoints might suggest a lower efficacy of vericiguat compared to sacubitril/valsartan (in PARADIGM-HF) or dapagliflozin (in the DAPA-HF trial), the annualized event rate for the primary endpoint was greater for the VICTORIA than for the PARADIGM-HF (4.2 events per 100 patient-years at risk vs. 2.7), and close to the rate observed in DAPA-HF (4.0 events per 100 patient-years). Even the benefit on first HF hospitalization was greater in VICTORIA than that in PARADIGM-HF (3.2 events per 100 patient-years vs. 1.6) and close to DAPA-HF (2.9 events per 100 patient-years) [5]. Nonetheless, these comparisons did not account for heterogeneity and uncertainty around point estimates and did not assess whether the differences in metrics of efficacy achieved statistical significance. A more sophisticated statistical approach as the network meta-analysis (NMA) provides the unique opportunity to compare multiple treatments using indirect comparisons across trials based on a common comparator [32–34]. Our NMA exploring the relative efficacy of sacubitril/valsartan, vericiguat, and SGLT2i did not indicate a significant superiority of one of the examined drug strategies, except for SGLT2i over vericiguat for the endpoint “HF hospitalization”. Specifically, therapy with dapagliflozin was not associated with a significant reduction in the risk of cardiovascular death or HF hospitalization and of cardiovascular death alone, as compared to sacubitril/valsartan and vericiguat, and with a trend toward reduced risk of HF hospitalization as compared to sacubitril/valsartan. Despite the mostly non-significant differences, dapagliflozin had a higher surface under the cumulative ranking (SUCRA) score (a synthetic measure of efficacy) than sacubitril/valsartan and vericiguat [35].

## Future perspectives

HF is characterized by a neurohormonal activation aimed at maintaining an adequate tissue perfusion mainly by improving haemodynamics by an increase in arterial pressure and plasma volume, and promoting cardiac remodelling. Although initially compensatory, these mechanisms are ultimately detrimental, fostering HF progression. The renin–angiotensin–aldosterone (RAAS) and sympathetic nervous systems (SNS), acting through the second messenger cyclic adenylate monophosphate (cAMP) at the cellular level, are key players in this pathophysiological context, and therapies targeting these systems (beta-blockers, ACEi/ARBs, MRAs) represent the standard of care for HFrEF [1].

The NO-sGC-cGMP pathway acts as a counterregulatory axis in HF. In particular, NPs counteract the effects of RAAS and SNS by promoting sodium and water excretion and inhibiting cardiac and vascular remodelling by activating cGMP signalling, which, in turn, can reduce cAMP by activating the phosphodiesterase 2 isoform [36]. As a consequence, approaches aimed at potentiating cGMP signalling might act synergistically to the current standard of care of SNS and RAAs inhibitors. Among therapies increasing cGMP, only sacubitril/valsartan and vericiguat improved patient outcome over ACEi/ARB, beta-blockers, and MRA in HFrEF [26, 27, 37–44]. In particular, vericiguat was evaluated in patients with recently decompensated HF and a high risk of cardiac events, reducing the composite of cardiovascular death and first HF hospitalization over the standard of care. This result was driven by a reduction in HF hospitalizations [9]. Based on preliminary data, the prognostic benefit from vericiguat does not seem to be associated with reverse remodelling [45], contrary to sacubitril/valsartan [41]. The VICTORIA trial was underpowered to assess the effects of combined vericiguat and sacubitril/valsartan therapy. Future research should try to elucidate whether vericiguat or sacubitril/valsartan is more effective compared to the combination of ACEi/ARB, beta-blockers, and MRA in patients with chronic HF. Moreover, the combination of sacubitril/valsartan and vericiguat deserves consideration as NPs act through pGC, while vericiguat potentiates sGC activity and response to NO. Cardiomyocyte response to sacubitril/valsartan or vericiguat might be additive or even synergic, given that pGC and sGC have different efficacy in terms of PKG activation under baseline conditions and during adrenergic stimulation [42].

SGLT2i have been recently added to the armamentarium of therapies for stable HFrEF following the striking results in terms of reduced cardiovascular mortality and HF hospitalizations of DAPA-HF and EMPEROR-REDUCED trials [5, 6]. Although the main apparent target of SGLT2i is the kidney, a direct effect on the heart (e.g., an interaction with the Na^+^/H^+^ exchanger 1 at cardiomyocyte level) is likely based on preclinical studies [43]. Given that the postulated mechanisms of action of SGLT2i and other neurohormonal antagonist/modulators, such as sacubitril/valsartan and vericiguat, are distinct, a possible additive effect of these drug classes cannot be excluded. Indeed, subgroup analyses of DAPA-HF and EMPEROR-REDUCED trials showed that SGLT2i beneficial effect on outcome was maintained in patients on sacubitril/valsartan (representing about 11% and 19% of the whole cohort, respectively, in the 2 trials) [5, 6]. Based on these premises, some authors have proposed the combination of beta-blockers, ACEi/ARB/ARNI, MRA, and SGLT2i as the new mainstay of treatment for HFrEF [44]; nonetheless, more data are needed to confirm this assumption. On the other hand, no study has currently investigated the additive effect of vericiguat and SGLT2i, or even the combination of the 2 on top of an optimal medical therapy including sacubitril/valsartan. In terms of relative efficacy, our NMA showed that the effect of these 3 drug classes on outcome (cardiovascular death and HF hospitalization) is similar, with the only exception of SGLT2i being superior to vericiguat in terms of risk of HF hospitalization [35]. These results nonetheless do not exclude an additive effect of the 3 drug classes. Moreover, it must be pointed out that, compared to PARADIGM-HF and DAPA-HF, the VICTORIA trial enrolled more fragile patients (older, more symptomatic, with higher NP levels) who had recently recovered from an AHF event; therefore, vericiguat could be a valuable adjunct in this vulnerable group [9]. Accordingly, the recently published update of the Canadian Cardiovascular Society (HF) guidelines suggests considering vericiguat for HFrEF patients just recovered from HF hospitalization on top of a therapy including a beta-blocker, an ACEi/ARB/ARNI, a MRA and a SGLT2i [45].

**Fig. 1:**
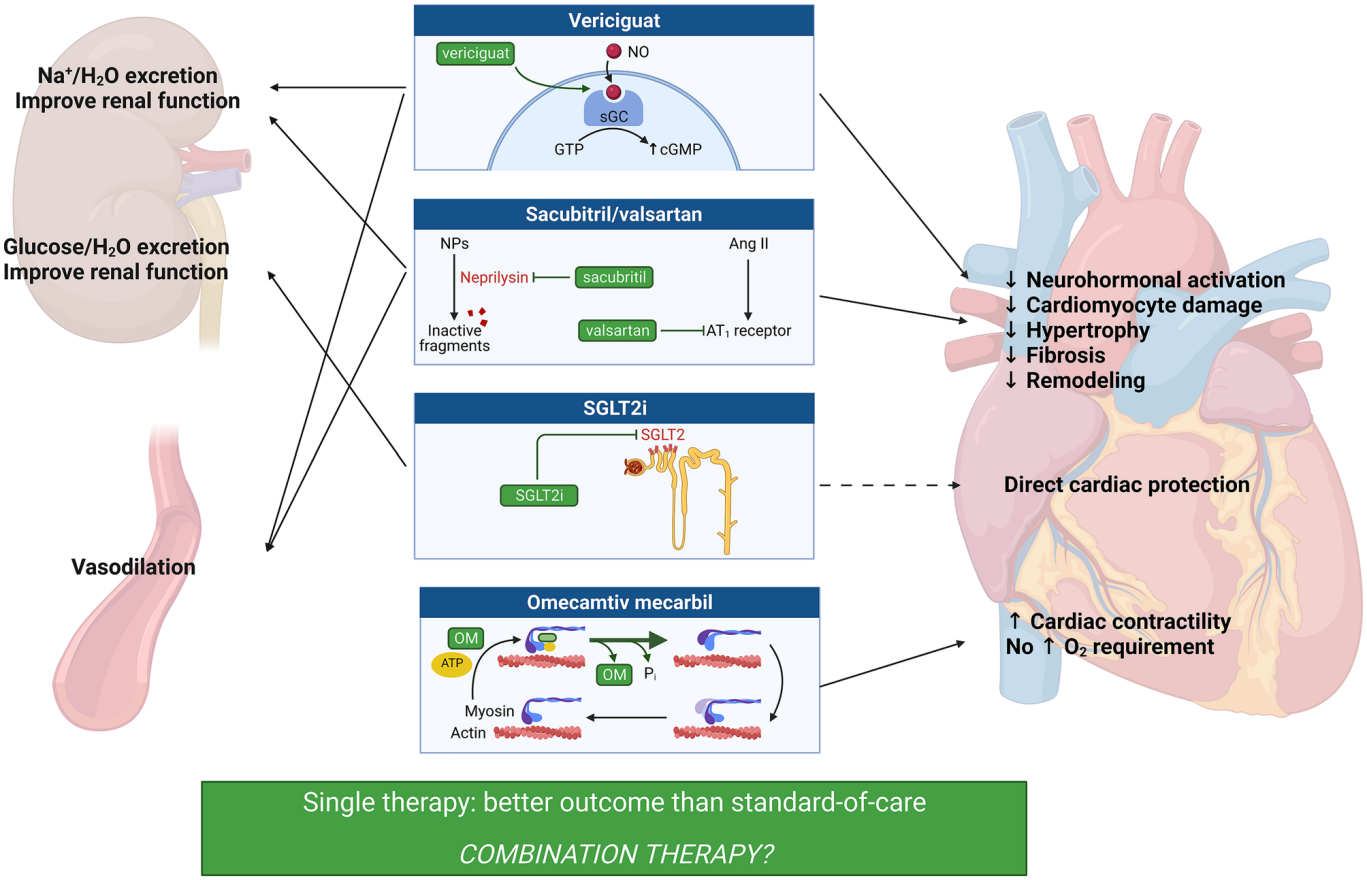
Novel therapies for heart failure, their mechanisms of action and final effects. See text for details. Ang, angiotensin; ATP, adenosine triphosphate; cGMP, cyclic guanosine monophosphate; GTP, guanosine triphosphate; NO, nitric oxide; NP, natriuretic peptide; OM, omecamtiv mecarbil; sGC, soluble guanylate cyclase; SGLT2i, sodium-glucose cotransporter 2 inhibitors

LV pump failure is a main feature of HFrEF; however, until recently, no orally taken drug directly acting on cardiac inotropism was available for chronic HFrEF, with the sole exception of beta-blockers. Omecamtiv mecarbil is a novel selective cardiac myosin activator that directly increases LV systolic function. The GALACTIC-HF trial randomized 8256 patients with stable LVEF ≤ 35% to either omecamtiv mecarbil or placebo, demonstrating a modest beneficial effect of this drug on the composite endpoint of time to first HF event (hospitalization or urgent visit) or death from cardiovascular causes, without significant differences regarding the separate components of the primary endpoint or the KCCQ score. Notably, about 19% of patients enrolled in this trial were on sacubitril/valsartan and < 3% on a SGLT2i, without significant results at the subgroup analyses [8]. Given the modest effect of omecamtiv mecarbil in the whole cohort, no definite conclusion can be made whether this drug class will ever enter the clinical stage and, therefore, whether a combination with other treatments for HFrEF should be even taken into consideration (Fig. 1).
